# The Relationship Between Smoking and Delayed Cerebral Ischemia After Intracranial Aneurysm Rupture: A Systematic Review and Meta-Analysis

**DOI:** 10.3389/fneur.2021.625087

**Published:** 2021-03-26

**Authors:** Xiaolong Ya, Chaoqi Zhang, Shuo Zhang, Qian Zhang, Yong Cao, Shuo Wang, Jizong Zhao

**Affiliations:** ^1^Beijing Tiantan Hospital, Capital Medical University, Beijing, China; ^2^Beijing Neurosurgery Research Institute, Beijing Tiantan Hospital, Capital Medical University, Beijing, China

**Keywords:** smoking, delayed cerebral ischemia, intracranial aneurysm, rupture, meta-analysis

## Abstract

**Background:** Delayed cerebral ischemia (DCI) is the main cause of death and disability after intracranial aneurysm rupture. Previous studies have shown that smoking can lead to DCI after intracranial aneurysm rupture. However, some recent studies have shown that nicotine, as the main ingredient of tobacco, can cause cerebral vasodilation. This view has led to a debate about the relationship between smoking and DCI. This study aims to determine the relationship between smoking and DCI.

**Methods:** A systematic literature search was performed according to PRISMA guidelines. The Cochrane Library, Web of Science, PubMed, and Embase online databases were searched for studies published up to September 2020. All studies related to smoking and DCI were included in the analysis. The R and RevMan software were used for data analysis, and random or fixed model analysis was selected depending on the degree of heterogeneity. Publication bias was examined by using the Begg–Mazumdar test and using contour-enhanced funnel plots with trim method.

**Results:** A total of eight original articles (12 cohorts) with 10,722 patients were included in this meta-analysis. There were statistically significant higher rates of DCI in the smoking group than in the non-smoking group (RR_total_ = 1.16, 95%CI: 1.05–1.27). After heterogeneity among cohorts was removed by sensitivity analysis, there was still a statistically significant difference in the incidence of DCI between the smoking and non-smoking groups (RR_total_ = 1.13, 95%CI: 1.07–1.20).

**Conclusions:** Although the effects of nicotine as the main component of tobacco are unclear in terms of cerebral vessels, the present study suggests that smoking is a risk factor for DCI in patients with ruptured aneurysm.

## Introduction

The prevalence of intracranial aneurysms is approximately 2.8%, and the annual rupture rate ranges within 0.5–0.2%. Although its incidence and annual rupture rate are not very high, the mortality and disability rate can reach as high as 30% once a rupture occurs (1–3). Delayed cerebral ischemia (DCI) caused by cerebral vasospasm is the main cause of poor prognosis after intracranial aneurysm rupture (4, 5). Most scholars have considered that the occurrence and severity of DCI after intracranial aneurysm rupture mainly depend on the volume of subarachnoid hemorrhage (SAH) (6–8). However, some studies have revealed that the occurrence of DCI is also affected by many other factors. Smoking is quite common among people with aneurysmal SAH (aSAH), and this has always been considered by many scholars as an important influencing factor of the process for intracranial aneurysm (including aneurysm growth, rupture, recurrence after treatment, and DCI) (9–12). However, in recent years, some studies have shown that nicotine is the main ingredient of tobacco and that instead of causing cerebral vasospasm, this would promote cerebral vasodilation (13, 14). This view has made a confusion on whether smoking is a risk factor of DCI and has led to a debate on the relationship between smoking and DCI. In addition, if smoking is a risk factor for DCI, cessation would be an important method to reduce the burden of DCI after SAH. The present meta-analysis aims to determine the relationship between smoking and DCI.

## Materials and Methods

### Inclusion and Exclusion Criteria

The inclusion criteria are as follows:

(1) both prospective and retrospective cohort studies;
(2) studies related to smoking and DCI after aneurysm rupture; and
(3) data about smoking and DCI that have integrity or could be calculated from the article.

The exclusion criteria are as follows:

(1) non-human studies;
(2) studies on DCI other than intracranial aneurysms;
(3) studies related to DCI without the smoking factor;
(4) articles classified as abstracts, letters, editorials, expert opinions, reviews, case reports, or laboratory studies;
(5) studies without sufficient data for analysis; and
(6) duplicate articles or data.

### Search Strategy

We searched the Cochrane Library, PubMed, Embase, and Web of Science for cohort studies analyzing the relationship between smoking and DCI published up to September 2020. Our search terms and procedures were as follows: (1) “smoke” OR “smoking” OR “cigar*” OR “tobacco” OR “nicotine”; (2) “intracranial” OR “cerebral” OR “brain” OR “intracerebral” OR “cranial”; (3) “vasospasm*” OR “angiospasm” OR “spasm.” The retrieval formula was as follows: (1) AND (2) AND (3). Two investigators who received normative and unitive training independently screened the titles and abstracts of each study after duplicate references were excluded. Following our initial screening, full texts were obtained for all studies with the potential to meet our minimum inclusion criteria.

### Data Extraction and Quality

Two assessors independently evaluated the quality of all included studies using the nine-star Newcastle–Ottawa Scale (NOS) (15). The NOS scores of each study are shown in Table 1. Studies were judged according to the three aspects of NOS evaluation: selection, comparability, and outcome between the smoking and non-smoking groups. A study with a NOS score ≥6 is considered good quality. All these data were analyzed using RevMan software (version 5.4 for windows).

**Table 1:** The characteristics of included studies for the present meta-analysis.

**Study (author/year)**	**Study design**	**Participants**	**Age (mean ± SD)**	**Female (n%)**	**Smoking rate**	**Smoking status**	**DCI rate**	**Missing data**	**NOS score**
NASAH (10), 1992–1994	Prospective cohort study	902	50.4 ± 11.8	68%	62.4%	Current smoking	56.8%	0	6
EASAH (10), 1991–1993	Prospective cohort study	1,023	50.4 ± 11.2	67%	61.2%	Current smoking	43.8%	0	6
CANADA (10), 1990–1992	Prospective cohort study	242	49.9 ± 11.0	63%	68.2%	Current smoking	54.1%	3	6
NICSAH I (10), 1987–1989	Prospective cohort study	904	48.9 ± 11.3	64%	59.2%	Current smoking	56.2%	2	6
NICSAH II (10), 1989–1991	Prospective cohort study	365	50.5 ± 11.3	68%	55.1%	Current smoking	53.7%	0	6
Hormuzdiyar (9), 2016	Retrospective cohort study	5,784	54.9 ± 13.7	68.4%	31.1%	Current or former smoking	15.9%	700	5
Hubert (12), 2018	Retrospective cohort study	463	56.0 ± 13.2	70.2%	48%	Current smoking	21%	11	7
Todd (11), 1997	Prospective cohort study	70	49.8 ± 13.6	63%	64.3%	History of smoking	28.6%	0	7
Stefan (16), 2009	Retrospective cohort study	163	55.5 ± 17.2	63%	66%	History of smoking	21.5%	0	6
Tetsuji (17), 2014	Retrospective cohort study	350	–	–	40%	Current or former smoking	26%	20	6
Maimaitili (18), 2016	Retrospective cohort study	343	53	61.2%	18.7%	Current or former smoking	53.1%	110	5
Sushant (19), 2013	Retrospective cohort study	108	53 ± 12.3	71.3%	36.1%	History of smoking	38%	0	7

The same reviewers extracted all study data and effect estimates for the relationship between smoking and DCI; all disagreements were discussed, and a final decision agreed to by both reviewers. Other study-related outcomes, including publication year, sample size, study design, mean age (±SD), smoking rate (%), DCI rate (%), and female rate (%) were extracted using a standardized form (Table 1). All these data were analyzed using R software (version 4.0.3 for windows).

### Publication Bias Analysis

Finally, publication bias was assessed by contour-enhanced funnel plots and combined with the trim method. The Begg–Mazumdar test is used to detect whether the funnel plot is symmetric. If the shape of funnel plots was symmetric, we considered that there was no obvious publication bias. Otherwise, we will further determine that whether there were some publication biases using the contour-enhanced combine with the trim method. The publication bias analyses were performed by R software according to standard statistical procedures (20).

### Statistical Analysis

In this meta-analysis, the relationship between smoking and DCI was measured by estimating the relative risk (RR) with its 95% confidence interval (CI). Statistical heterogeneity was assessed using *I*^2^, a random effect model was used for analysis if the *I*^2^ was >50%, and a sensitivity analysis was further performed. For analysis with an *I*^2^ <50%, a fixed-effect model was used, and a sensitivity analysis was not performed. Significance was set at *p* < 0.05.

## Results

### Study Selection and Characteristics

**Figure 1:**
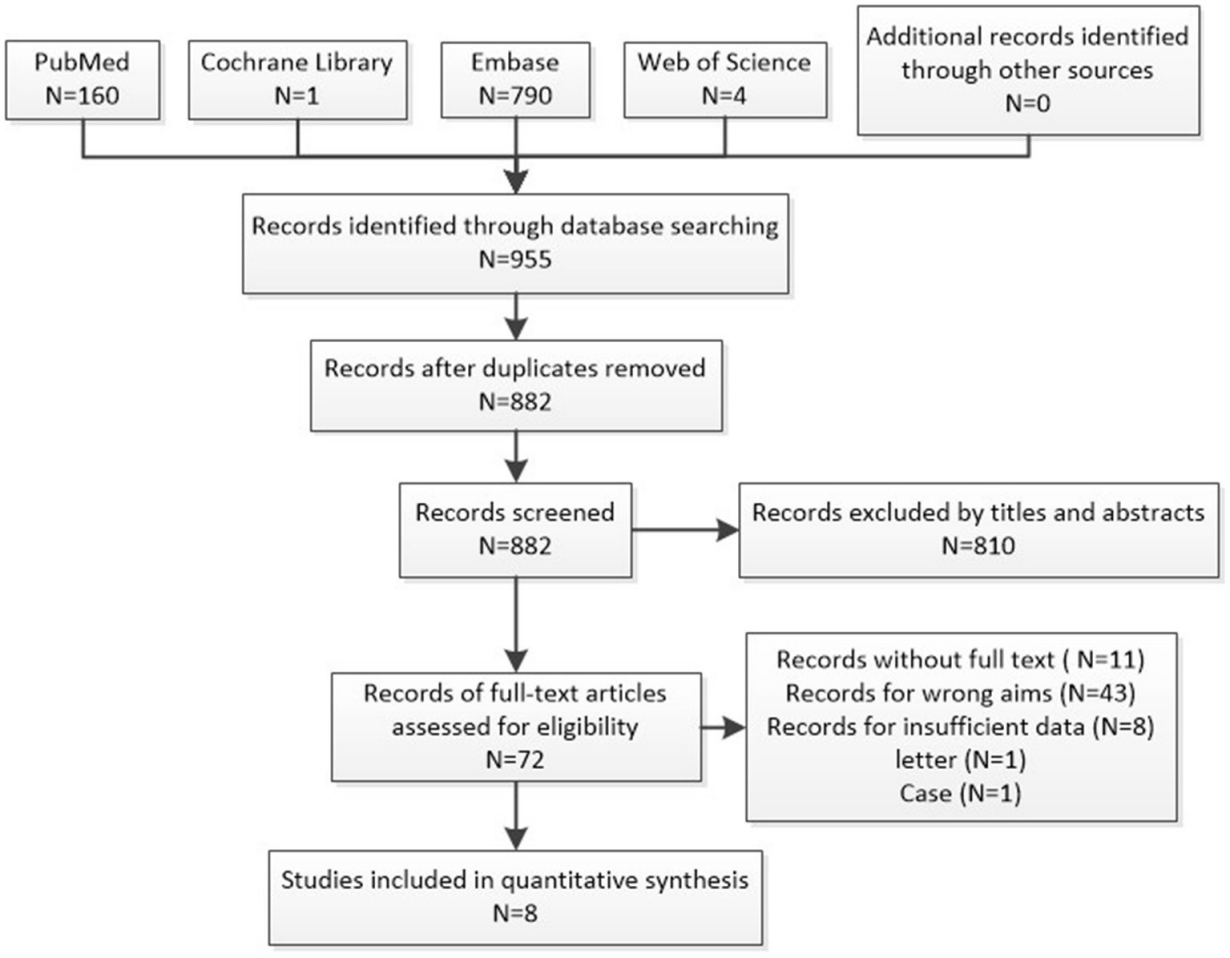
Flow diagram of literature search and selection of included studies for meta-analysis.

We found a total of 955 results, in which 882 records remained after removal of duplicates. After screening the title and abstracts, 765 records were excluded, leaving 72 eligible studies. The full texts of 11 studies cannot be obtained. Full texts were obtained for the remaining 61 studies, of which 53 were excluded (43 studies for wrong aims, 8 studies for insufficient data, 1 for letter article, and 1 for case report), yielding a final set of 8 literatures (12 cohorts) covering 10,722 participants, of which 4,499 (42%) were smokers, with 1,646 showing DCI after ruptured intracranial aneurysms, and 6,223 (58%) were non-smokers, with a 25% incidence of DCI. Out of these studies, two were prospective cohort studies, and six were retrospective cohort studies. The study selection process is shown in Figure 1, and the basic characteristics of the included cohorts are shown in Table 1.

### Association Between Smoking and DCI Estimated With Relative Risk

**Figure 2:**
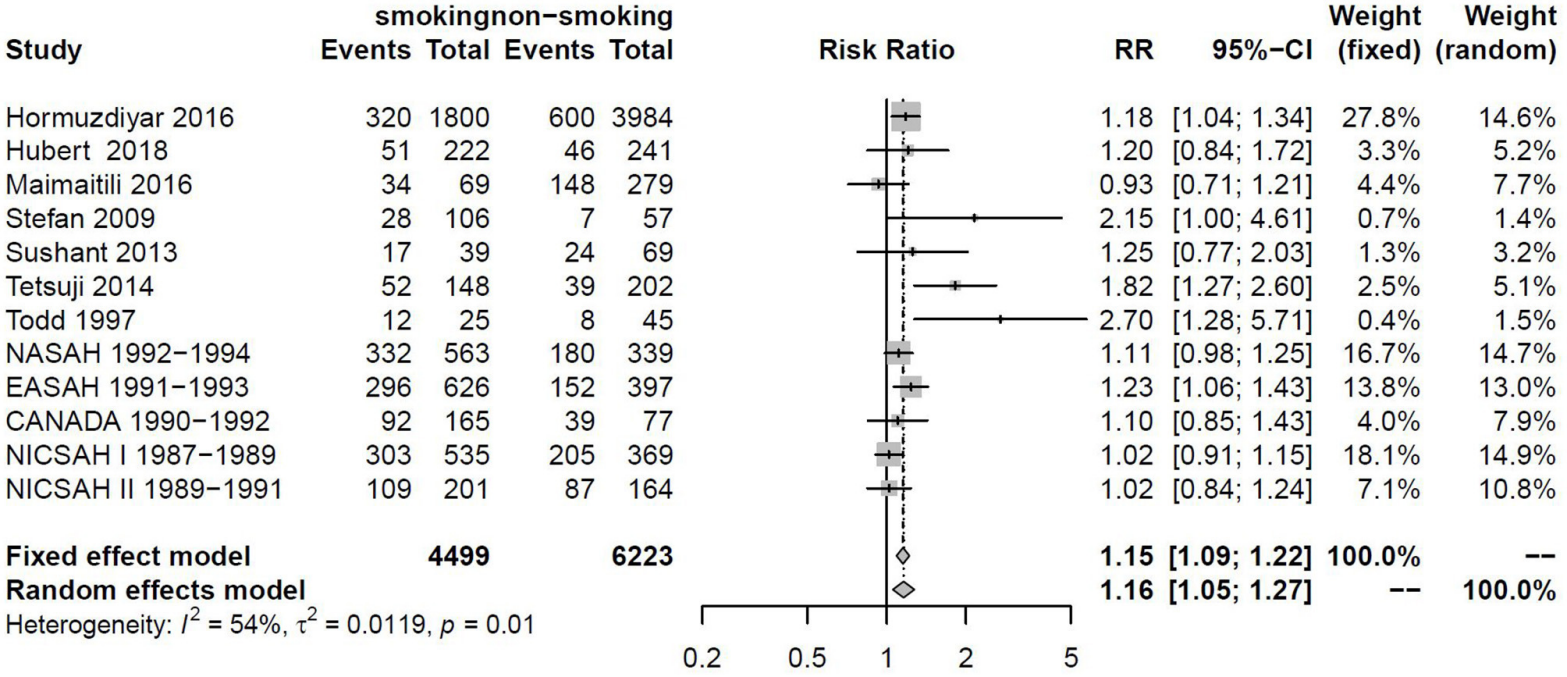
Forest plot of the smoking as one of the risk factors for DCI.

**Figure 3:**
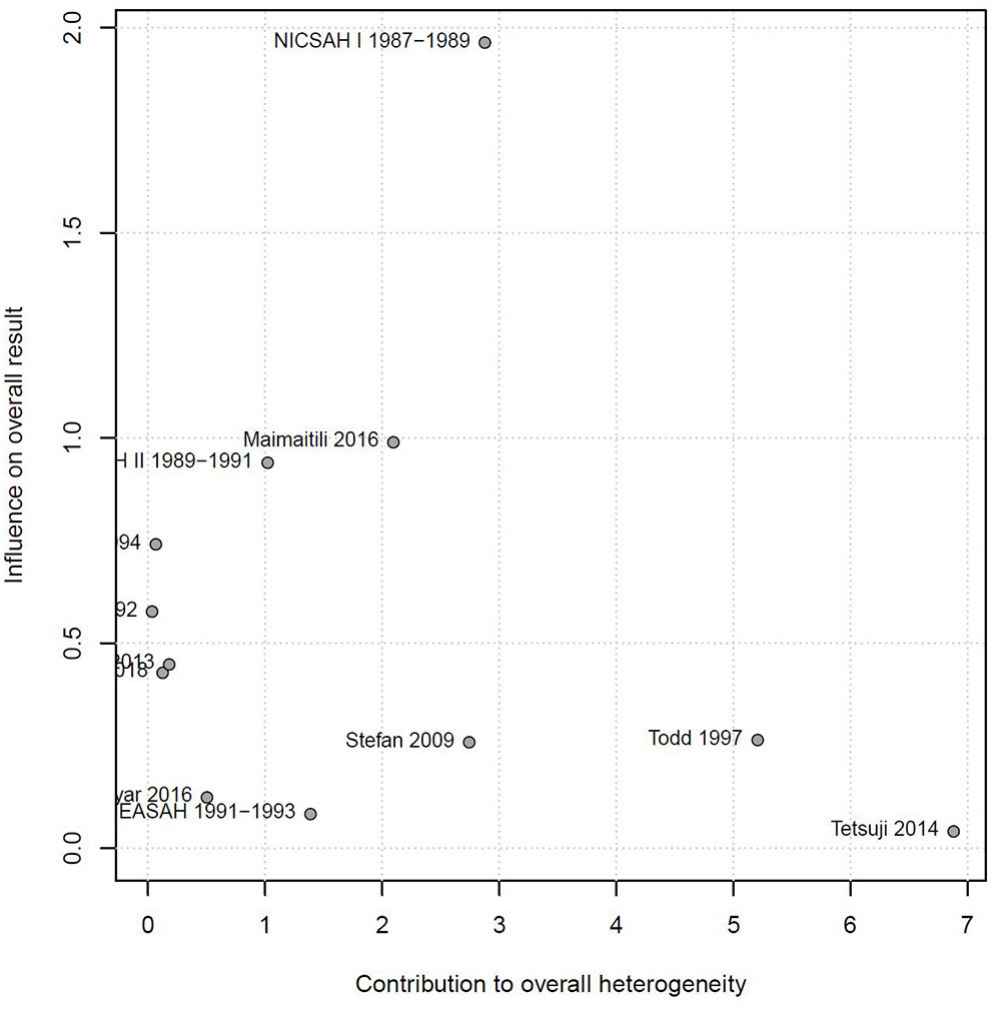
Baujat graph shows the source of heterogeneity.

The relationship between smoking and DCI was assessed based on relative risk. There were statistically significant higher rates of DCI in the smoking group and non-smoking group (RR_total_ = 1.16, 95%CI: 1.05–1.27). This analysis was estimated using a random-effect models, as significant heterogeneity was found between studies (*p* = 0.01 <0.05, *I*^2^ = 54% > 50%). These data were shown in Figure 2. The Baujat graph performed by R software showed that the heterogeneity of this analysis mainly comes from the study of Todd and Tetsuji (Figure 3).

### Subgroup Analysis by Smoking Status

**Figure 4:**
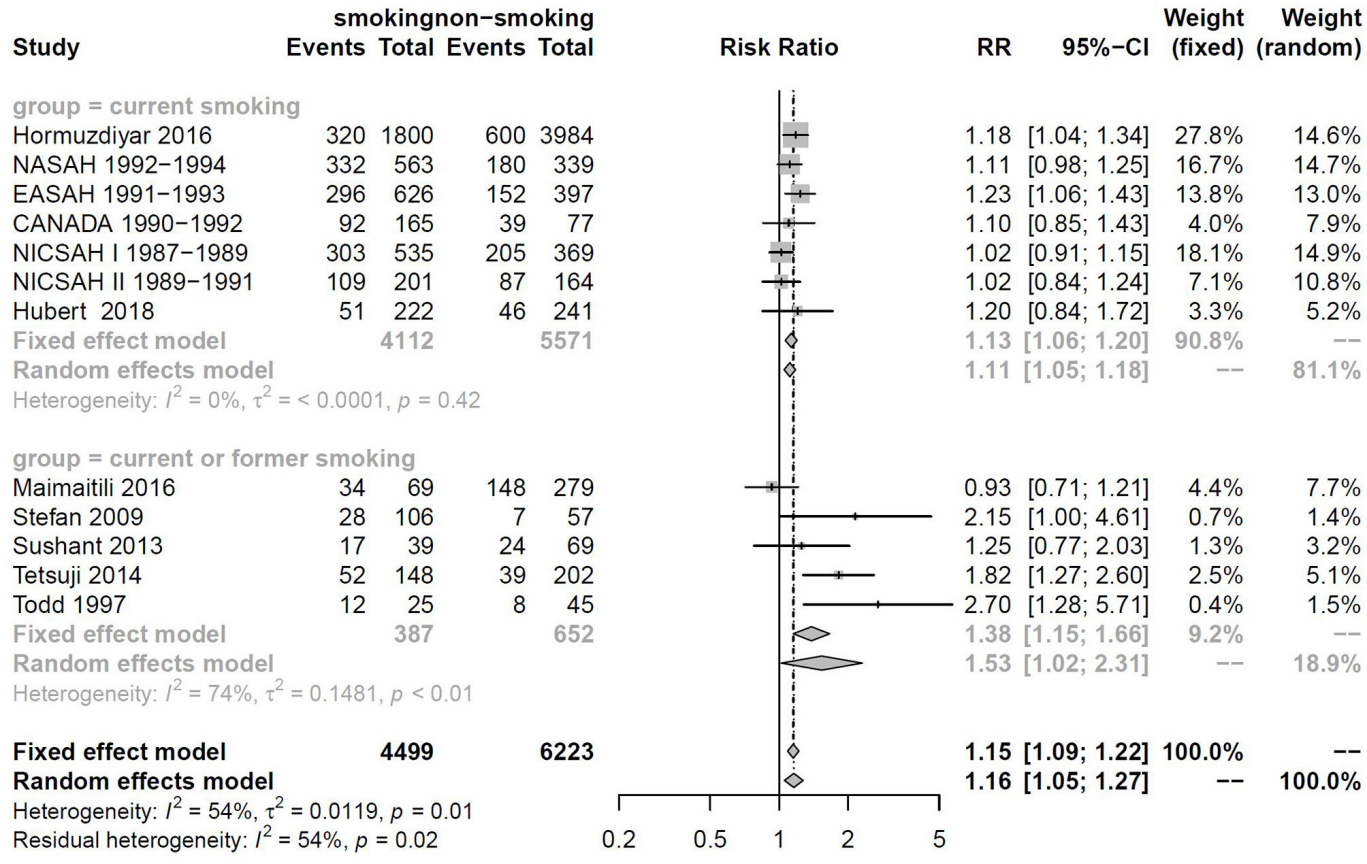
Forest plot of subgroup analysis by smoking status.

The descriptions of smoking status were not consistent among included studies, and therefore, these studies were subdivided into “current smoking” and “current or former smoking” for subgroup analysis. There were six cohorts with 9,683 participants included in the current smoking subgroup, while 1,036 participants were included in the current or former smoking subgroup. DCI rates for the current smoking and current or former smoking groups were 29 and 36%, respectively. RRtotal values for the current smoking and current or former smoking groups were 1.13 (95%CI: 1.06–1.20, *I*^2^ = 0% < 50%, *p* = 0.42) and 1.53 (95%CI: 1.02–2.31, *I*^2^ = 54% > 50%, *p* = 0.02), respectively (Figure 4).

### Subgroup Analysis by the DCI Measurement Method

There are many methods to the detection of vasospasm. The sensitivity of these detection methods may be different. Among included studies, Stefan, Sushant, and Todd clearly stated that only transcranial Doppler (TCD) was used as a method to diagnose DCI, Maimaitili and Tetsuji stipulated that digital subtraction angiography (DSA) was used as a method to monitor TCD, while other studies use multiple means to monitor DCI. Because the data required for subgroup analysis could not be extracted, only two studies using DSA as a monitoring means were excluded. The remaining studies were divided into “multiple measurements” and “TCD measurement.”

**Figure 5:**
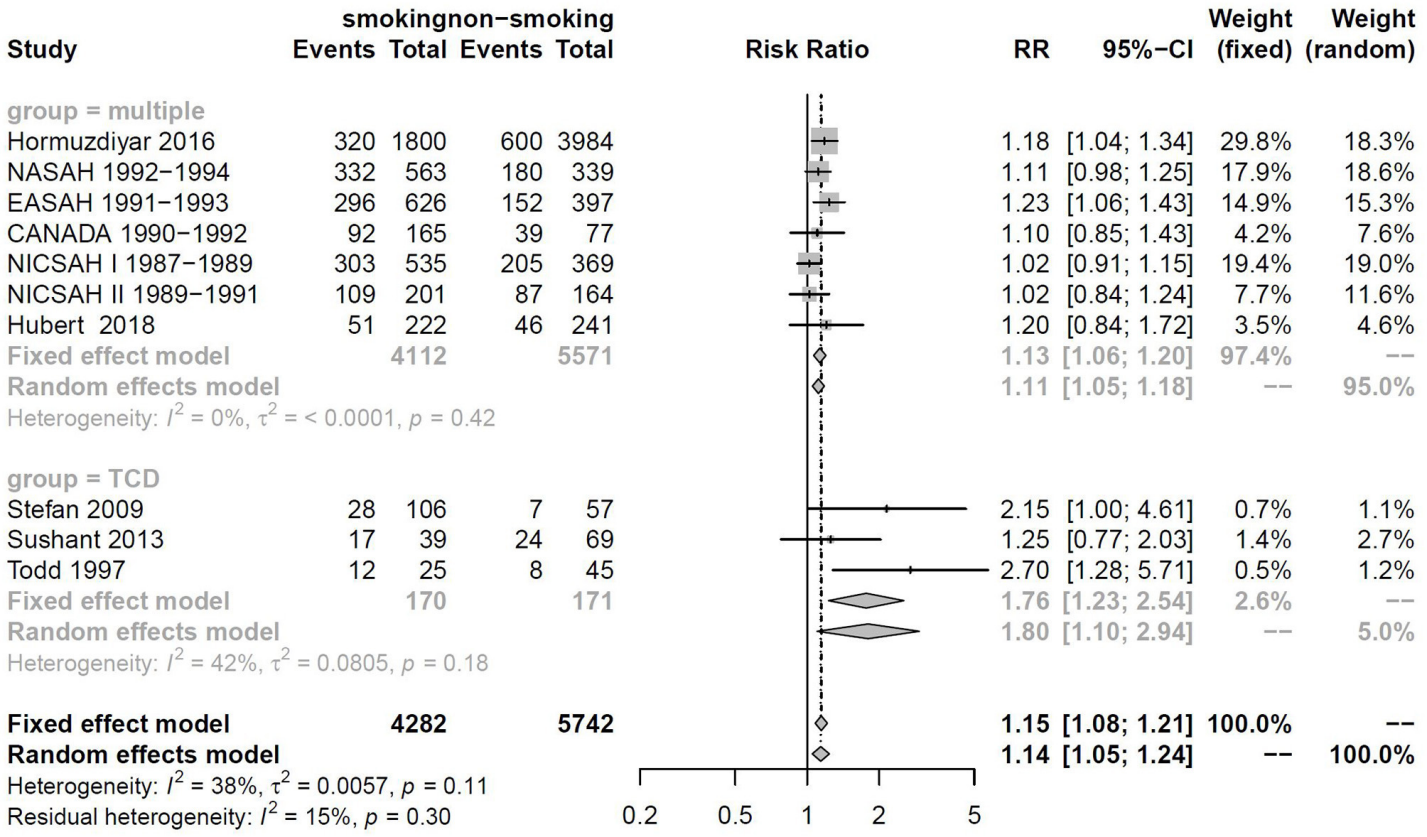
Forest plot of subgroup analysis by the DCI measurement method.

RRtotal values for the “multiple measurements” and “TCD measurement” groups were 1.13 (95%CI: 1.06–1.20, *I*^2^ = 0% <50%, *p* = 0.42) and 1.76 (95%CI: 1.23–2.54, *I*^2^ = 42% < 50%, *p* = 0.18), respectively (Figure 5).

### Sensitivity Analysis

**Figure 6:**
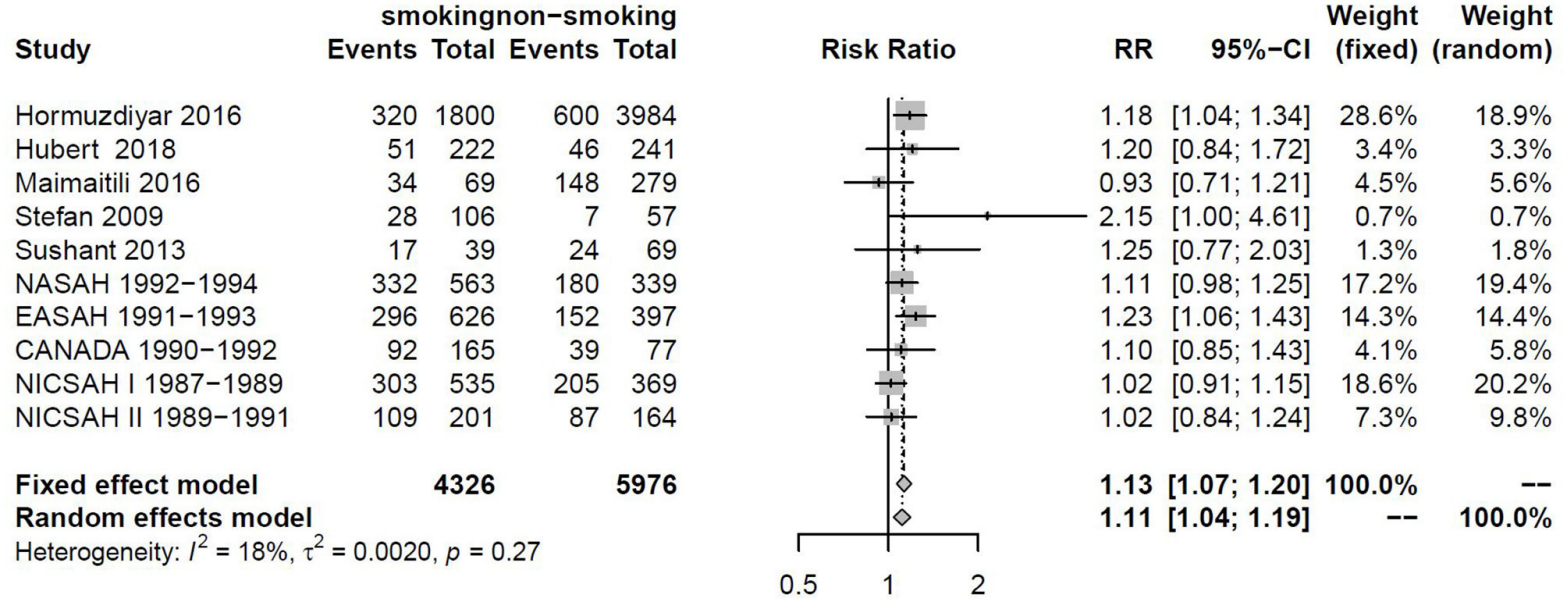
Forest plot of smoking as one of the risk factors for DCI after removing heterogenous cohorts by sensitivity analysis.

Sensitivity analysis also found that the cohort data of Todd and Tetsuji were the main sources of heterogeneity. These studies were removed, and there was no statistical heterogeneity among these cohorts (*I*^2^ = 18% < 50%, *p* = 0.27 > 0.1). This meta-analysis was calculated again using fixed-effects models and showed that smoking remained a risk factor for DCI (RRtotal = 1.13, 95%CI: 1.07–1.20) (Figure 6).

### Quality Assessment

As shown in Table 1, there were three studies with a NOS score of 7, seven studies with a NOS score of 6, and two studies with a NOS score of 5. Based on these assessments, 83.3% of studies met the standard for good quality of the investigators (Table 2 for NOS score).

**Table 2:** NOS scores for included studies.

**Study (author/year)**	**Selection**	**Comparability**	**Exposure**	**Quality scores**
NASAH (10), 1992–1994	***		***	6
EASAH (10), 1991–1993	***		***	6
CANADA (10), 1990–1992	***		***	6
NICSAH I (10), 1987–1989	***		***	6
NICSAH II (10), 1989–1991	***		***	6
Hormuzdiyar (9), 2016	****		^*^	5
Hubert (12), 2018	***	^*^	***	7
Todd (11), 1997	***	^*^	***	7
Stefan (16), 2009	***		***	6
Tetsuji (17), 2014	***		***	6
Maimaitili (18), 2016	***		**	5
Sushant (19), 2013	***	^*^	***	7

* *The score of each cohort on each item of the NOS scale*.

**Figure 7:**
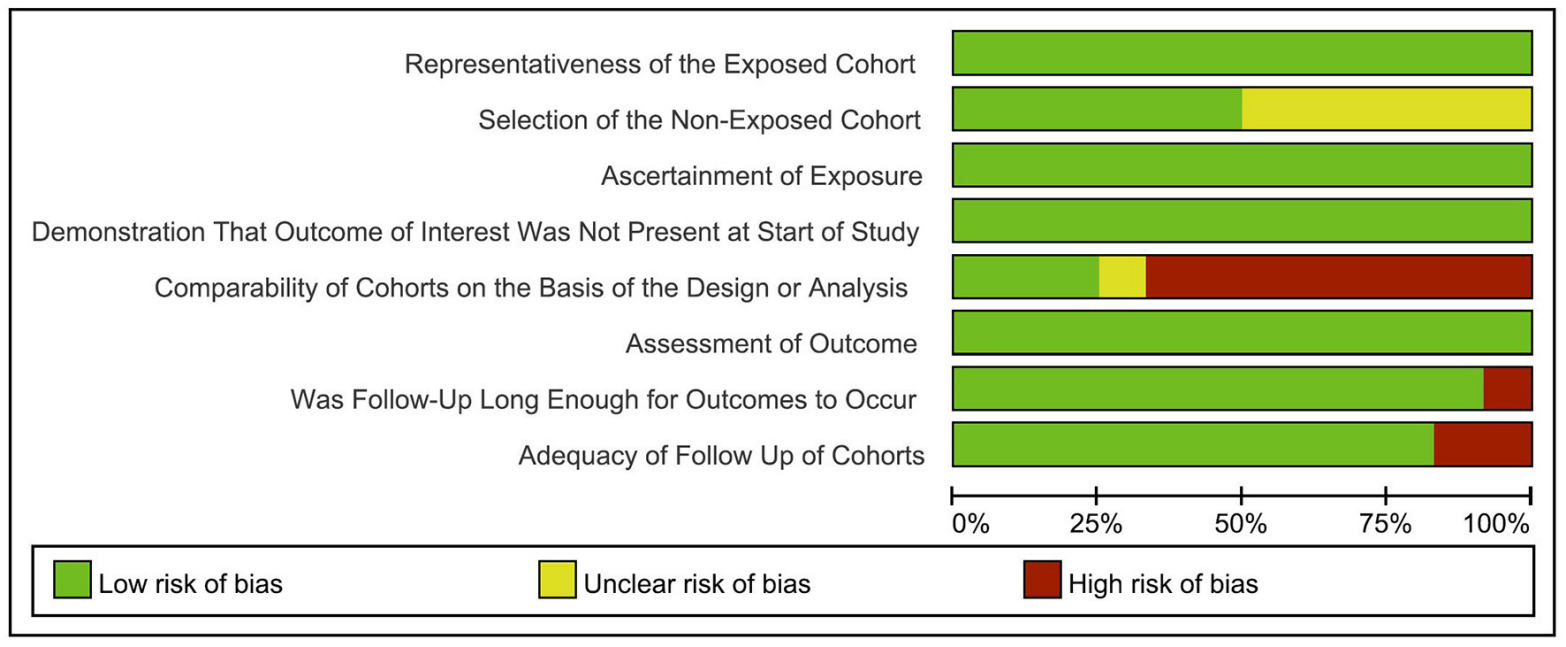
Risk-of-bias graph: review authors' judgements about each risk of bias item presented as percentages across all included studies.

**Figure 8:**
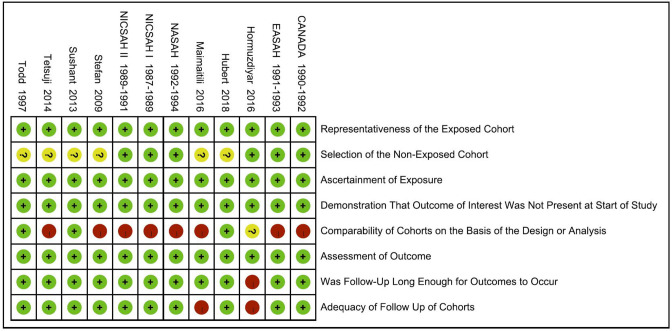
Risk-of-bias summary: review authors' judgements about each risk-of-bias item for each included study.

Next, a risk-of-bias graph was generated for each of the included studies. The risk of bias for each cohort was presented as a percentage across all included studies (Figure 7), as well as individually (Figure 8). Good quality was strongest in “selection,” which included the items of representativeness of the exposed cohort and selection of the non-exposed cohort. Comparability of outcome issues was deemed to be a risk of bias in these studies. Other factors between the exposure group and control group were not controlled due to the different research purposes in these studies. Unclear risks of bias were observed for the criteria “Demonstration That Outcome of Interest Was Not Present at Start of Study.” Most patients developed DCI during hospitalization. Hence, there was no significant risk of bias, in terms of “Was Followed-Up Long Enough for Outcomes to Occur” and “Adequacy of Follow Up of Cohorts.”

### Publication Bias

**Figure 9:**
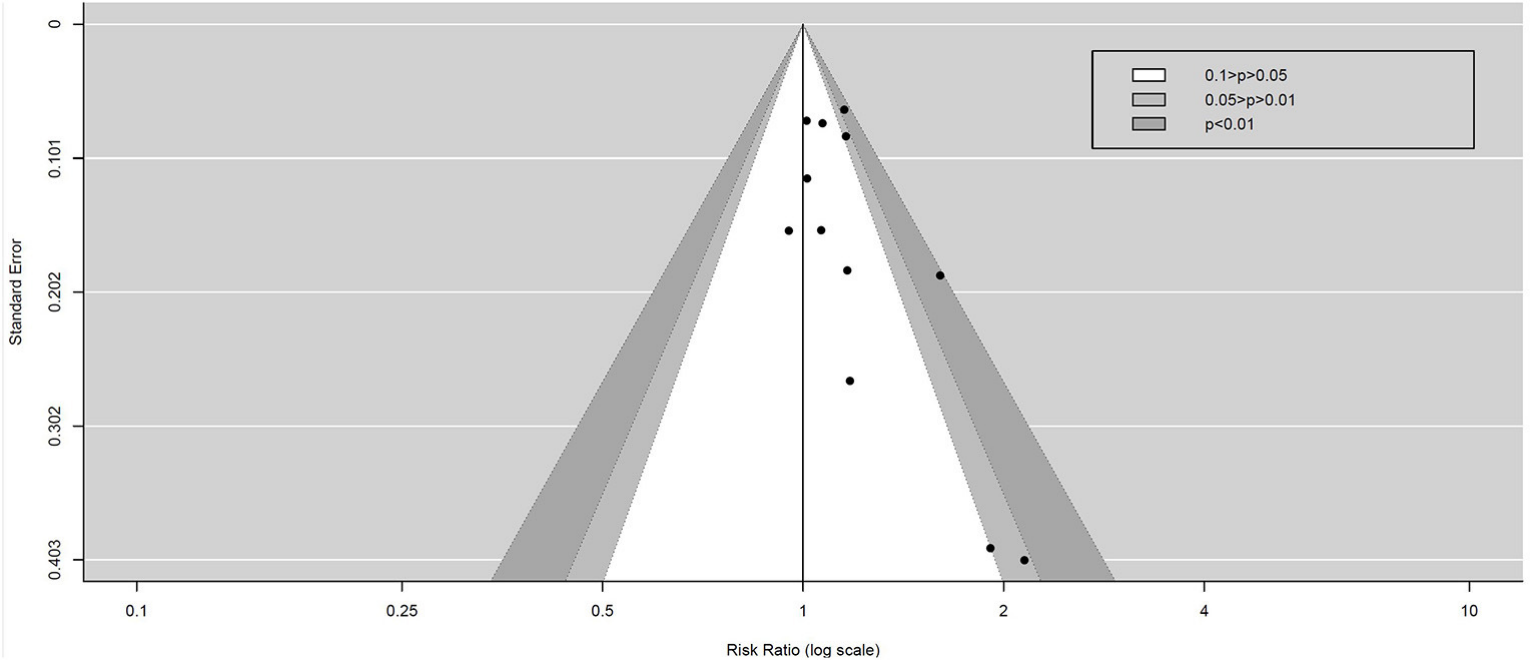
Contour-enhanced funnel plot assessed the potential publication bias.

**Figure 10:**
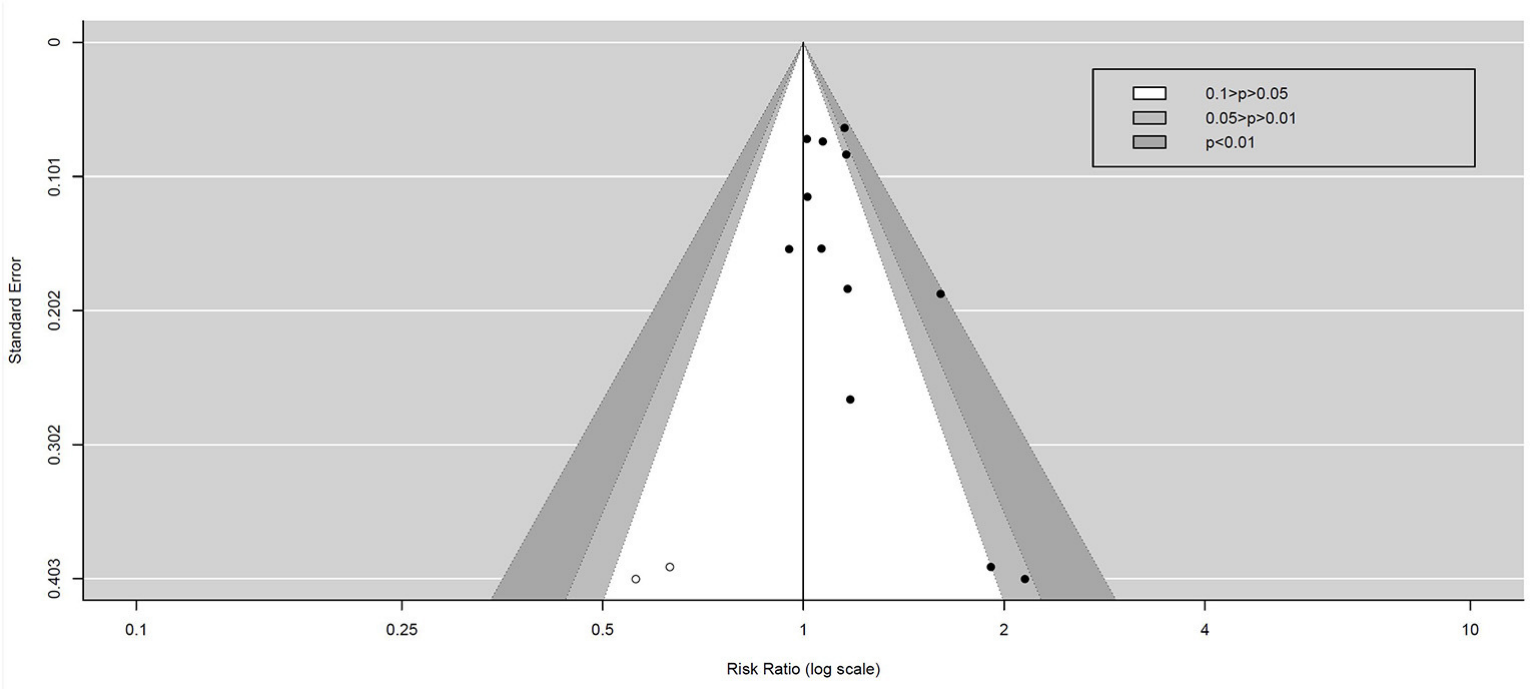
Contour-enhanced funnel plot combined with trim method.

Contour-enhanced funnel plots were generated to assess the potential publication bias among the included studies, as shown in Figure 9. The Begg–Mazumdar test indicates that there was an asymmetry of this funnel plot (*Z* = 2.0572, *p* = 0.039 < 0.05). Contour-enhanced funnel plots combined with the trim method show that these potentially unpublished studies do not affect the final result, as shown in Figure 10.

It can be observed from this image that for some additional studies (the hollow circle in the picture), there is a need to correct the asymmetry of the funnel plot. However, these additional studies will still distribute in non-statistically significant areas (white areas), indicating the existence of non-statistically significant unpublished studies.

## Discussion

Smoking is one of the harmful behaviors, and many diseases, especially cardiovascular and cerebrovascular diseases, are related to this. Many previous studies have shown that smoking is an important risk factor for the growth and rupture of intracranial aneurysms. Approximately 29% of the attributable risks of SAH are due to smoking (21–23). However, the influence of tobacco exposure on outcome after SAH has not been thoroughly investigated, and conflicting results have been reported (24–26). Therefore, the impact of smoking on clinical vasospasm, which is the leading cause of morbidity and mortality after the initial SAH, remains controversial (10, 11, 27). The majority of scholars have considered that smoking can increase the incidence of DCI and the mortality and disability after the intracranial aneurysm ruptures (10, 11, 16, 17). However, some researchers have reported that nicotine replacement therapy in smokers who sustained aneurysmal SAH was associated with superior outcomes. Nicotine replacement therapy should be appropriately performed to reduce the incidence of withdrawal symptoms, which would lead to a poor prognosis (28, 29). In addition, some animal experiments have revealed that nicotine, which is the main ingredient of tobacco, instead of causing cerebral vasospasm, would promote cerebral vasodilation (13, 30, 31). These results have led to the confusion on whether smoking is a risk factor of DCI, leading to a debate on the relationship between smoking and DCI. Considering that the mechanism of the smoking effect on cerebral vasculature remains unclear, relevant studies were pooled together to determine whether smoking would trigger DCI after aneurysm rupture.

After numerous studies, at present, tobacco exposure has become widely accepted as a risk factor for the development and rupture of intracranial aneurysms (32–34). However, the influence of tobacco exposure on DCI after SAH is still not well-understood (24–26). Previous studies did not reveal the clear association between smoking and vasospasm (35–37). However, since the study conducted by Lasner, several studies have reported that cigarette smoking is an independent predictor of symptomatic vasospasm after aSAH (9–11, 38). It remains difficult to consider that smoking before SAH may increase the risk of symptomatic vasospasm several days after SAH. Furthermore, the molecular pathophysiological mechanisms for cerebral arterial spasm are also not completely understood. Some scholars have considered that tobacco may increase the effect of substance P, thereby triggering NO-dependent (nitric oxide-dependent) vasodilation. Furthermore, this may also release NO to relax the cerebral blood vessels through the central acetylcholine neuron (14). Another part of these scholars consider that tobacco can increase the activity of protein kinase C by damaging the vascular endothelium, thereby inducing vasoconstriction (13). In combining the outcome data obtained from 12 cohorts, the present meta-analysis suggests that smoking is still a risk factor for DCI, regardless of whether this was based on a subgroup analysis or comprehensive analysis. Among smokers, DCI has a higher incidence. It is known that cigarette smoke contains more than 4,000 chemical substances. Some of the substances may promote contraction of the cerebral blood vessels, while others may promote relaxation. The effects of these substances are either superimposed or offset at the same time, and these may also occur in chronological order. Briefly, the effect of smoking on cerebral blood vessels may be a complex interactive process (31). Recently, the study conducted by Maurizio provides a new way of thinking. That study revealed that the stiffness index is higher in patients with DCI, representing an independent predictor of DCI in patients with SAH (39). In addition, Robert's meta-analysis results suggested that nicotine can increase the stiffness of arterial walls (40). Although the effect of nicotine on vasospasm remains controversial, this may also indirectly cause DCI after SAH by affecting the stiffness of the vascular wall. Tseng's summary of the treatment of DCI with statins also supports this view (41). Regardless of whether acute or chronic smoking may make the blood vessels produce a state of tension, resulting in increased vascular hardness, this state may be a pre-spastic state. This may be because this effect is only the initial state of DCI. A few days after the occurrence of SAH, the balance between local diastole and contraction is broken under the conditions of red blood cell decomposition and local inflammatory reaction, which finally leads to the occurrence of DCI. Based on the results of the present study, smoking remains a risk factor for DCI. It is a necessary measure to strengthen the monitoring of DCI in smokers with SAH. Furthermore, timely and active clinical intervention measures for patients with DCI may be able to reduce the mortality rate it causes, to some extent.

The present study had some limitations. First, all of the included studies were observational and would have inevitable observation bias. Second, the DCI diagnostic criteria and assessment of smoking status varied across studies. DCI was defined as the vasoconstriction found by many different imaging methods after neurological deterioration. Different imaging detection methods might have deviations in accuracy. The tobacco exposure status was also not consistent. Some studies used current smoking as an exposure factor, and other studies also included former smokers into the exposure group. To some extent, these factors might increase the heterogeneity among included studies. Finally, we used meta regression analysis to find that age is also a source of heterogeneity (τ^2^ = 0.0546, *p* < 0.0001). Because relevant data could not be extracted from included literatures, no subgroup analysis was performed. These unclarified factors and other unmeasured or unidentified confounding factors also affect the credibility of these research results.

## Conclusion

Although the effects of nicotine, which is the main component of tobacco, remain unclear, in terms of cerebral vessels, smoking is still a risk factor for DCI in patients with a ruptured aneurysm. Limited by the number and quality of included studies, a more dedicated and well-controlled cohort study is warranted for the further evaluation of the relationship between smoking and DCI.

## Data Availability Statement

The original contributions presented in the study are included in the article/supplementary material, further inquiries can be directed to the corresponding author/s.

## Author Contributions

XY had the idea for the article, performed the data analysis, and drafted this article. CZ performed the literature search. SW, YC, QZ, and JZ critically revised the work. JZ as study supervision approved the final version of the manuscript on behalf of all authors. All authors contributed to the article and approved the submitted version.

## Conflict of Interest

The authors declare that the research was conducted in the absence of any commercial or financial relationships that could be construed as a potential conflict of interest.
